# Telemedicine Complements Effective Health-Care Delivery but Is Not a Panacea

**DOI:** 10.1371/journal.pmed.0030367

**Published:** 2006-08-29

**Authors:** Rajiv Sarin

**Affiliations:** ACTREC, Tata Memorial Centre, Kharghar, Navi Mumbai, India

Like most health researchers in India, I share Sanjit Bagchi's enthusiasm and optimism about the potential of telemedicine in improving health-care delivery in our diverse country [1]. However, it is important to maintain a balanced perspective and debunk overstatements such as those used by Bagchi in his conclusion “This potential was well summed up by Dr. Devi Shetty: ‘In terms of disease management, there is [a] 99% possibility that the person who is unwell does not require [an] operation. If you don't operate you don't need to touch the patient. And if you don't need to touch the patient, you don't need to be there. You can be anywhere, since the decision on healthcare management is based on history and interpretation of images and chemistry … so technically speaking, 99% of health-care problems can be managed by the doctors staying at a remote place—linked by telemedicine.’”

Maybe Devi Shetty, well known in telemedicine circles, has been quoted out of context, but it is not evident as such. In the wider context of the story, such conclusions as “If you don't operate you don't need to touch the patient”; “And if you don't need to touch the patient, you don't need to be there”; and “so technically speaking, 99% of health-care problems can be managed by the doctors staying at a remote place” are misleading. In the near future, online physicians or health professionals cannot replace the onsite ones for even 30% of all health problems, let alone 99% as pointed out by Shetty. Even for providing essential and readily accessible health services, we need to augment the number of onsite physicians and other health professionals with improved skill-mix, more pragmatic public health policies, and enabling of telemedicine. Though India has been a pioneer in evaluating telemedicine for improving health-care delivery [2,3], its rightful place can be established only through major coordinated research efforts [4].

Free-access medical journals are a forum for debate on emerging health issues and have the responsibility to implement rigorous peer review and editorial control to prevent wide dissemination of any misleading statements or misquotes, as the case may be. Sweeping concluding statements from opinion leaders appearing in respectable journals may unduly influence those lacking insight in the challenges of health-care delivery in underserved regions and allow further propagation by medical journalists living in virtual reality.

To realize its full potential, we need to identify more innovative applications of telemedicine and generate robust data on its cost-effectiveness in different health-care settings [4]. But first we need changes in skill-mix [5], which includes enhancement of skills among various staff groups, role substitution between different groups, delegation up and down a uni-disciplinary ladder, and innovation in roles. Until we have telemedicine models that are universally implementable, with proven cost-effectiveness and user satisfaction [6], we need a stance of cautious optimism.
